# Factors influencing institutionalization of health technology assessment in Kenya

**DOI:** 10.1186/s12913-023-09673-4

**Published:** 2023-06-22

**Authors:** Rahab Mbau, Anna Vassall, Lucy Gilson, Edwine Barasa

**Affiliations:** 1 Department of Global Health and Development, London School of Hygiene and Tropical Medicine, Keppel Street, London, WC1E 7HT UK; 2 Health Economics Research Unit, KEMRI Wellcome Trust Research Programme, 197 Lenana Place, P.O. BOX 43640-00100, Nairobi, Kenya; 3 Health Policy and Systems Division, School of Public Health and Family Medicine, University of Cape Town, Anzio Road 7925, Cape Town, South Africa; 4 Centre for Global Health and Tropical Medicine, Nuffield Department of Medicine, University of Oxford, Roosevelt Drive, Oxford, OX3 7LG UK; 5 Institute of Healthcare Management, Strathmore University, Karen Ole Sangale Road, P.O. BOX 59857-00200, Nairobi, Kenya

**Keywords:** Health technology assessment, Institutionalization, Kenya

## Abstract

**Background:**

There is a global interest in institutionalizing health technology assessment (HTA) as an approach for explicit healthcare priority-setting. Institutionalization of HTA refers to the process of conducting and utilizing HTA as a normative practice for guiding resource allocation decisions within the health system. In this study, we aimed to examine the factors that were influencing institutionalization of HTA in Kenya.

**Methods:**

We conducted a qualitative case study using document reviews and in-depth interviews with 30 participants involved in the HTA institutionalization process in Kenya. We used a thematic approach to analyze the data.

**Results:**

We found that institutionalization of HTA in Kenya was being supported by factors such as establishment of organizational structures for HTA; availability of legal frameworks and policies on HTA; increasing availability of awareness creation and capacity-building initiatives for HTA; policymakers’ interests in universal health coverage and optimal allocation of resources; technocrats’ interests in evidence-based processes; presence of international collaboration for HTA; and lastly, involvement of bilateral agencies. On the other hand, institutionalization of HTA was being undermined by limited availability of skilled human resources, financial resources, and information resources for HTA; lack of HTA guidelines and decision-making frameworks; limited HTA awareness among subnational stakeholders; and industries’ interests in safeguarding their revenue.

**Conclusions:**

Kenya’s Ministry of Health can facilitate institutionalization of HTA by adopting a systemic approach that involves: - (a) introducing long-term capacity-building initiatives to strengthen human and technical capacity for HTA; (b) earmarking national health budgets to ensure adequate financial resources for HTA; (c) introducing a cost database and promoting timely data collection to ensure availability of data for HTA; (d) developing context specific HTA guidelines and decision-making frameworks to facilitate HTA processes; (e) conducting deeper advocacy to strengthen HTA awareness among subnational stakeholders; and (f) managing stakeholders’ interests to minimize opposition to institutionalization of HTA.

**Supplementary Information:**

The online version contains supplementary material available at 10.1186/s12913-023-09673-4.

## Background

Health systems resource constraints and continued resource wastage have led to growing interest in explicit healthcare priority-setting processes to inform universal health coverage (UHC)-related decisions [1, 2]. Explicit healthcare priority-setting processes are deliberative, evidence-based, inclusive, systematic, and transparent processes for informing resource allocation decisions [1]. An example of an explicit healthcare priority-setting approach is health technology assessment (HTA). HTA is “*a multidisciplinary process that uses explicit methods to determine the value of a health technology to inform decision-making towards an equitable, efficient and high-quality health system*” [3]. A health technology is any intervention that can promote health; prevent, diagnose, or treat disease; prolong lives; or inform health service delivery. Examples include diagnostic tests, medicines, vaccines, procedures (medical and surgical), policies, and programs [3, 4].

With the ever-growing demand for health technologies arising from UHC commitments, advancements in scientific knowledge, larger older population groups and rising burden of communicable and non-communicable diseases, the need for HTA to inform explicit healthcare priority-setting becomes more crucial as health systems budgets remain limited [5–7]. Integrating HTA into healthcare priority-setting processes is a good governance measure that strengthens health systems by promoting transparency, inclusivity, and accountability in decision-making through systematic, deliberative, and inclusive processes [8]. HTA also promotes good governance by providing policymakers with an efficient means of allocating resources thus promoting sustainability in resource limited health systems striving to achieve UHC [7].

The impact and sustainability of HTA as an approach for explicit priority-setting in healthcare is dependent on its institutionalization [9, 10]. Institutionalization of HTA refers to the process of conducting and utilizing HTA as a normative practice for guiding healthcare priority-setting processes [10]. This requires development of institutional and organizational structures and processes that produce and utilize HTA in decision-making [9, 10]. In countries where HTA has been institutionalized, it is routinely conducted as a way of informing health policy decisions on:- (a) development and revision of health benefits packages for pharmaceutical and non-pharmaceutical products; (b) development of clinical guidelines; (c) market authorization of health technologies; and, (d) pricing and reimbursement regulations for health technologies [8–10].

There are more high- and upper-middle-income countries that have institutionalized HTA as an explicit approach for healthcare priority-setting than low and lower-middle-income countries particularly in Sub Sahara Africa [11, 12]. Literature shows that institutionalization of HTA is affected by factors that may be context or country-specific [13–15]. Examining and identifying which country-specific factors are influencing institutionalization of HTA is important as it enables policymakers and technocrats to introduce appropriate measures to address them [13–15]. However, studies examining factors that influence institutionalization of HTA in low and lower-middle income countries remain limited as shown in a recent scoping review in which only 23% of the 77 articles described experiences in low and lower-middle income countries [16].

Kenya- a lower-middle-income country in Sub-Sahara Africa- has embarked on institutionalizing HTA. In 2018, Kenya’s Ministry of Health introduced a reform on explicit healthcare priority-setting process for health benefits package which mandated the development of a framework for institutionalizing HTA in Kenya. Previously, only a few studies have examined the status of HTA in Kenya. These studies showed that there was limited availability of HTA guidelines [17] and human resource capacity [11, 17, 18], and limited formal utilization of HTA [17, 18]. Given the recent reform in Kenya, we conducted the following study to identify factors that were influencing institutionalization of HTA in this context.

## Methods

### Study design

We conducted a qualitative case study on the institutionalization of HTA in Kenya.

### Conceptual framework

**Fig. 1:**
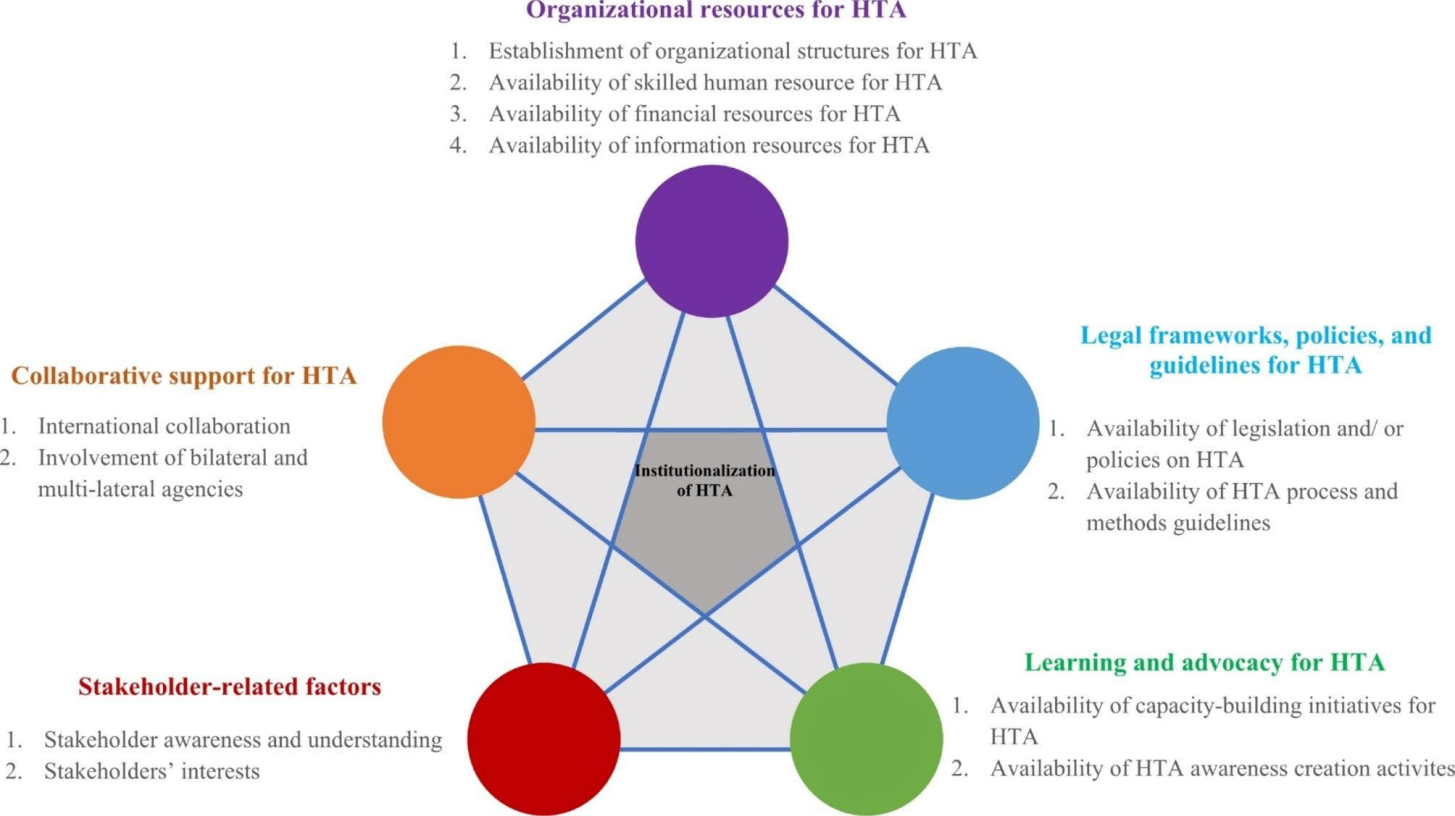
A conceptual framework on factors influencing institutionalization of HTA

We used a conceptual framework developed from a scoping review of 77 empirical articles on factors influencing institutionalization of HTA across 135 countries of different income levels globally [16]. Majority of the retrieved articles- 77% (n = 59) - described factors influencing institutionalization of HTA in high and upper-middle income countries. Based on this review, we identified five sets of factors that influenced a country’s capacity to conduct and utilize HTA as a way of allocating resources in the health sector. These factors included: -a) organizational resources for HTA; b) legal frameworks, policies, and guidelines for HTA; c) learning and advocacy for HTA; d) stakeholder-related factors; and e) collaborative support for HTA. These factors were complexly interlinked as presented in the conceptual framework on Fig. 1. This interlinkage meant that the factors could influence each other. We utilized this framework to develop questions for our data collection tool, to generate themes during data analysis, and to synthesize findings from the data.

### Study setting

We conducted the study in Kenya which has a population of approximately 53.8 million people [19]. Kenya has a devolved governance system with administrative, fiscal, and political roles split among one national and forty seven county governments [20]. Health system functions are also devolved within this two-tiered governance system. Within the national government, the Ministry of Health (MOH) is the highest decision-making body for the health sector. The MOH is responsible for building capacity, developing health policies for the health sector, overseeing service delivery in national referral healthcare facilities, and providing technical assistance at the national and county level [20]. Within the county governments, the County Departments of Health are responsible for implementing national health policies and overseeing service delivery in county healthcare facilities such as primary healthcare facilities (community units, dispensaries and health centres) and secondary healthcare facilities (primary and secondary referral hospitals) [20].

### Study population and sampling strategy

We used purposive and snowballing techniques to sample participants. The aim was to obtain rich descriptions of the case study by involving knowledge-rich participants. The purposive criterion was a participant’s known involvement in activities related to the institutionalization of HTA in Kenya. The purposively selected participants were subsequently asked to identify other participants who were active in the HTA institutionalization space. We stopped sampling at saturation, that is, when no new information was emerging from additional interviews [21]. We interviewed 30 stakeholders (Table 1). We do not provide any demographic information to preserve the anonymity and confidentiality of the study participants.

**Table 1:** List of participants

Category	Number
Development Partners	n = 6
Local research and academic organizations	n = 6
Ministry of Health (MOH)	n = 8
Semi- Autonomous Government Agencies	n = 10
**Total**	**30**

### Data collection methods

We used in-depth interviews and document reviews to collect data between January and April 2022.

#### In-depth interviews

We requested participants to engage in the study via telephone or email- none refused participation. Participants reviewed the study’s information sheet and provided informed consent. We conducted interviews directly via face-face or remotely via zoom videoconferencing at a time of convenience to the participant. Interviews were guided by a semi-structured interview guide (see Additional File 1), and they lasted between 25 and 80 min. All interviews were recorded using an encrypted audio recorder.

We also took fieldnotes during interviews to identify points that needed further clarification and to summarize emerging themes. We linked each fieldnote to the respective interview using the same identifier. Following the interviews, fieldnotes were transferred to Microsoft word to prevent data loss.

#### Document reviews

We reviewed various documents as shown in Table 2. These documents included organizational and media reports with relevant information on institutionalization of HTA. We identified these documents from study participants, online searches, and two members of the study team who had previously been involved in HTA-related activities in Kenya. We conducted interviews and document reviews simultaneously to enable triangulation of data from each of these data sources.

**Table 2:** List of documents reviewed

Types of documents	Examples
Government documents (national policies and laws)	• Health Act 2017 • Health Products and Technologies Supply Chain Strategy 2020–2025 • Kenya Health Policy 2014–2030 • Kenya Health Sector Strategic and Investment Plan 2013–2017 • Drafts of the Kenya Health Financing Strategy 2015–2030
Local Research Organizations’ documents	• Reports on stakeholder engagement workshops • PowerPoint presentations made during stakeholder workshops • Reports on HTA capacity in Kenya
Health Benefits Package Advisory Panel’s (HBPAP) documents	• Final Report of the Universal Health Coverage Health Benefits Package Advisory Panel Report on the study visit by the Health Benefits Package Advisory Panel to Thailand on HTA • HBPAP reports and annexes • HBPAP PowerPoint presentations
Development partners’ reports	• Mission report on Health Benefits Package Advisory Panel Study visit to Thailand • Japan International Cooperation Agency Loan policy action on HTA • Mission report on National Hospital Insurance Fund Health Financing Reforms Experts Panel visit to Thailand on UHC and HTA
Media reports	• Web media e.g., Development Partners websites and MOH websites • News media e.g., Online newspaper reports • Social media e.g., Twitter

### Data analysis

We transcribed all audio-files verbatim. We then verified the quality of transcription by comparing each transcript to the respective audio-file. We uploaded all transcripts, field notes, and electronic documents to NVIVO Pro software (QSR International, Massachusetts) for effective organization during data analysis. We analyzed the data thematically using the Braun and Clarke 6-step approach [22]. In Step 1, we immersed ourselves in the data through reading and re-reading to familiarize ourselves with the contents of each data source. In Step 2, we identified and coded the data using a deductive approach by deriving the codes from the concepts outlined in the study’s conceptual framework. In Stage 3, we generated a list of themes by identifying recurrent and meaningful patterns within the coded data that were of relevance to the objective of the study. In Stage 4, we verified the quality of the themes by checking whether the themes reflected the patterns of meaning in the coded data across the interview transcripts and documents. In Step 5, we extracted quotes and excerpts that supported the identified themes. In Step 6, we synthesized the findings and linked the discussion of these findings to existing literature to produce this manuscript.

### Reflexivity

Two of the authors have participated in HTA-related processes in Kenya. This participation not only influenced their interest in studying HTA but also facilitated easier access to participants who were involved in the institutionalization process and to organizational documents that reported on the institutionalization process. These two authors were also professionally associated with KEMRI-Wellcome Trust which provided technical support for institutionalizing HTA in Kenya, and funding support for the study. This professional affiliation facilitated easier access to organizational documents. Potential bias arising from the positionality of these two authors was minimized by involving two other authors who did not play any role in HTA related activities in Kenya and who were not affiliated with KEMRI-Wellcome Trust.

### Trustworthiness

To build trustworthiness in our study findings, we employed the following strategies. Firstly, we triangulated study methods by collecting data using more than one data collection method, that is, by combining interviews and document reviews [23, 24]. Since interviews are prone to recall bias, we minimized this methodological shortcoming by supplementing interviews with document reviews which are good sources for retrieving accounts of past events [25, 26]. Secondly, we triangulated data sources by interviewing participants from different organizations to explore multiple perspectives hence reducing the risk of bias associated with using only one data source [23, 24]. Lastly, we held bi-weekly peer debriefing sessions during data collection and analysis to mitigate against any potential bias that may have otherwise been introduced by 2 of the authors from their previous involvement in HTA-related processes in Kenya. In these peer debriefing sessions, we not only critiqued the interview topic guide for the clarity of its contents but also the interview transcripts to ensure grounding of the findings in the data [23, 24].

## Results

The data shows that several factors were influencing institutionalization of HTA in Kenya as discussed below.

### Limited availability of organizational resources for HTA

#### Establishment of organizational structures for HTA

Since 2018, the MOH has established new organizational structures to conduct and utilize HTA, and to oversee implementation of HTA. This followed the Government’s prioritization of UHC in 2017 [27]. These organizational structures included the Health Benefits Package Advisory Panel (HBPAP), a HTA focal point, the Medicines Affordability Pricing Advisory Committee (MAPAC) and, a HTA technical working group.

HBPAP, a semi-independent panel, was established in 2018 to develop an essential and affordable health benefits package for UHC [28]. HBPAP used a HTA approach to develop a health benefits package for UHC. HBPAP also developed a draft framework for institutionalizing HTA in Kenya [29].

*‘The establishment of the Panel [HBPAP] was the first attempt to set up a government driven HTA system where they recognized the use of HTA mechanism in decision-making’* Participant 5, MOH

A focal point or office for HTA was created within the MOH in 2020. It was tasked with overseeing and coordinating HTA institutionalization activities within the country. According to participants, since its establishment, the HTA focal point has overseen several HTA capacity-building and advocacy creation activities.

*‘We applaud the ministry for creating a HTA office. This office has been responsible for helping stakeholders walk the journey towards implementing HTA’* Participant 8, Semi-autonomous government agency

MAPAC was established in 2021 to promote access, availability, and affordability of pharmaceutical products. To this end, MAPAC aims to use HTA to promote transparency of the healthcare priority-setting processes for medicines. MAPAC also aims to use HTA to regulate and negotiate pricing of medical products towards making them affordable [30].

*‘MAPAC was inaugurated to develop strategic interventions to bring down healthcare costs. One of the strategic interventions the group has identified is HTA which can promote price transparency and visibility to everybody’* Participant 1, Semi-autonomous government agency

Lastly, a HTA technical working group was established in 2021 to develop a HTA strategy for Kenya. This technical working group is an 18-member team comprising of technocrats from:- (a) the MOH such as the Department of Health Policy, Research and Development, Department of Health Products and Technology, Department of Health Financing, and UHC secretariat; (b) Semi-autonomous government agencies such as Pharmacy and Poisons Board and Kenya Medical Supplies Agency; and (c) local research organizations such as KEMRI-Wellcome Trust [31].

In addition to the creation of new organizational structures for HTA, participants reported that the existence of multiple organizations involved in the regulation, procurement and purchasing of health technologies offered an opportunity to institutionalize HTA to inform these functions. Examples of these organizations included: - (a) the Pharmacy and Poisons Board which would use HTA outputs/ recommendations to inform entry and distribution of health technologies by ensuring they are of good quality to be efficacious and safe; (b) the Kenya Medical Supplies Agency which would use HTA outputs to inform procurement of health technologies for government-owned healthcare facilities; and (c) purchasers such as the national government, the county government, and the National Health Insurance Fund which would use HTA outputs to inform purchase of health technologies for government-owned healthcare facilities.

*‘We have institutions in strategic positions that deal with regulation, procurement and purchasing. Their presence provides a very good opportunity for introducing HTA as a priority-setting mechanism for their functions’* Participant 4, Semi-autonomous government agency

#### Limited availability of skilled human resource for HTA

In Kenya, the number of human resources with the technical skills to conduct HTA remain limited. A landscape analysis conducted in 2019 on HTA capacity in Kenya showed that more than 65% of health sector organizations had less than 5 individuals with formal training in HTA-related subjects such as health economics, mathematical modelling, statistics, evidence synthesis and epidemiology [32]. In addition, the analysis showed that more than 70% of health sector organizations had less than 5 individuals with practical experience in conducting systematic reviews or meta-analyses, cost-effectiveness analysis, and budget impact analysis [32].

*‘We do not have many people with the technical skills required to conduct HTA. The country is still in its infancy stages with regards to skills in evidence synthesis and economic evaluation. This inadequate local capacity is a barrier towards HTA’* Participant 9, Semi-autonomous government agency

#### Limited financial resources for HTA

The Government of Kenya has historically underfunded research. For example, approximately 3.5% of the government’s health budget is allocated to research which accounts for less than 30% of the resources required [33]. As a result, more than 70% of funding for research is obtained from external sources such as donors [33]. Given the limited funding, participants reported that the MOH could not meet the costs of conducting HTA which undermined institutionalization of HTA in Kenya.

*‘The lack of financial resources is a big stumbling block for institutionalization of HTA. At the moment, a lot of research activities are donor funded’* Participant 2, local research organization

#### Limited availability of information resources for HTA

Limited availability of information resources for HTA was reported as another factor limiting institutionalization of HTA in Kenya. Participants reported that despite improvements in Kenya’s health management information system, completeness, and timeliness of data reporting at the facility, county, and national levels were still limited. They also reported that data on costs were not routinely reported, and databases across purchasers were poorly linked. All these factors undermined the availability and quality of data which limited the capacity to conduct HTA.

*‘HTA processes are data hungry. For example, we need a database of costs to conduct economic evaluation and budget impact analysis. However, we do not have such a database’* Participant 10, Semi-autonomous government agency

### Adequate availability of legal frameworks and policies, but limited availability of guidelines for HTA

#### Adequate availability of legal frameworks and policies on HTA

Several legislation and policies on HTA exist in Kenya. These documents recognize various organizational and institutional aspects of institutionalization of HTA as shown in Table 3. According to participants, the establishment of organizational structures for HTA was partly a fulfilment of these legislation and policies.

**Table 3:** Laws and policies on HTA

Examples of laws or policies	Aspects of HTA institutionalization recognized in the document	Document extract
Health Act 2017 [34]	• Recognizes the role of HTA in: - ✓ supporting financing decisions towards UHC ✓ Regulation (e.g., market approval) of health technologies following assessment by a technically competent organization • Recognizes priority-setting criteria for HTA namely safety and effectiveness	“The department of health shall ensure progressive financial access to universal health coverage by taking measures that include…health technology assessment” “Legislation under section 62 shall provide for the granting of marketing approval only by a technically competent body after appropriate assessment has established that such a product meets generally recognized standards” “Any medicine, vaccine or other health product and technology intended for sale to members of the public shall be eligible for licensing only if- a) after due assessment, it is found to achieve the therapeutic or the intended effect it claims to possess, or which may reasonably be attributed to it; b) it is sufficiently safe under the normal conditions of use”
Kenya Health Policy 2012–2030 [35]	• Recognizes the need for: - ✓ A national HTA mechanism for assessing new health technologies ✓ A national framework for regulating health technologies • Recognizes priority-setting criteria for HTA namely quality, safety, efficacy/ effectiveness, and affordability	“Establishing a national appraisal mechanism for health products and technologies. This will provide guidance on the clinical and cost-effectiveness of new health products, technologies, clinical practices, and interventional procedures.” “Putting in place a harmonized national regulatory framework for health products and technologies. This shall advance the quality, safety and efficacy/effectiveness based on sound science and evidence. The regulatory framework shall be autonomous in its operations and shall encompass human drugs; vaccines, blood, and its products; diagnostics, medical devices, and technologies”
Health Sector Strategic and Investment Plan 2013–2017 [36]	• Recognizes: - ✓ establishment of a national HTA mechanism for health technologies and a national framework for regulation of health products as priorities ✓ priority-setting criteria for HTA such as clinical-effectiveness, quality, safety, cost-effectiveness and, ethical and cultural considerations ✓ the role of HTA in developing essential medicines list and clinical guidelines	“Establishing a national appraisal mechanism for health products and technologies- Institutionalize Heath Technology Assessment (HTA) to guide evidence-based use of medical devices, diagnostics & health technologies” “Assessment of Health products and technologies (HPTs): assessment of clinical effectiveness in the context of the national healthcare system, including cultural and ethical considerations. HTA assessment provides evidence-based guidance on strengthening the development of the Essential Medicines List and Clinical Guidelines” “Regulation of Health Product and Technologies (HPTs): ensuring that all HPTs meet the established standards of quality, safety and efficacy/performance.”
UHC policy 2020–2030 [37]	• Recognizes: - ✓ the role of HTA in guiding investment decisions on health technologies and promoting their rational use	“The institutionalization of Health Technology Assessments (HTA) will assist in guiding investment in point of care diagnostics, basic equipment for primary care services, and implements for essential surgeries. HTA will also guide the cost-effective and appropriate use of medicines in the era of growing antimicrobial resistance.”
Kenya Health Financing Strategy 2020–2030 [38]	• Recognizes: - ✓ The need to create a Health Benefits and Tariffs Authority to host national study and research functions on HTA ✓ the role of HTA in informing investments on new health technologies, provider payment rates, and revising the UHC health benefits package	“The Ministry of Health will institutionalize the functions by establishing a Health Benefits and Tariffs Authority which will host the national study and research functions on healthcare financing and health technology assessment” ‘It will produce up-to-date evidence on the effects of medicines and the latest health technologies on health outcomes” “Conduct a health technology assessment to inform, in a timely manner, the review of the benefits package and payment methods”
Health Products and Technology Supply Chain Strategy [33]	• Recognizes the need for: - ✓ a HTA policy to support management (e.g., pricing and market authorization) of health products and technologies ✓ a national roadmap for institutionalization of HTA ✓ building HTA capacity at national government agencies and county governments involved in health products and technology supply chain ✓ increased involvement of stakeholders in the HTA process at the national and county government to create demand and use of HTA ✓ surveys to assess use of HTA in pricing and market authorization of health products and technologies	“Strategic Objective 1.8: Institutionalize Health Technology Assessment (HTA) in HPT management 1.8.1 Develop an overarching Health Technical Assessment policy for Kenya to cover all HPT. 1.8.2 Develop a national HTA roadmap. 1.8.3 Build capacity for HTA at national government HPT supply chain agencies and county governments. 1.8.4 Increase stakeholder involvement at public and private sector, and both levels of government throughout the HTA process to help capture and improve the create value and applicability of HTA. 1.8.5 Undertake surveys to assess utilization of HTA in HPT Supply Chain planning and decision-making such as pricing and, market authorization for HPT.”
Strategy for HTA in the Kenyan Health Sector [39]	• Recognizes: - ✓ The mandate of HTA in developing, revising, or updating the essential medicines list, essential medical devices list, benefit package for UHC, and the national vaccines list ✓ The mandate of HTA in price negotiation for medical devices, medicines, and vaccines ✓ The organizations whose decisions will be informed by HTA namely the Kenya Medical Supplies agency, the National Health Insurance Fund and, the MOH ✓ The organizational and institutional architecture for HTA in Kenya including their roles and professional composition to support the HTA functions of topic nomination, topic selection, assessment, appraisal, and decision-making. ✓ The priority-setting criteria for topic selection in HTA namely effectiveness and safety, burden of disease, severity of disease, equity, catastrophic health expenditure, congruence with existing priorities, health workforce requirements and service, health commodities, and technologies requirements ✓ The priority-setting criteria for assessment stage namely cost-effectiveness analysis and budget impact analysis.	“The HTA process in Kenya will inform the following decisions: •The development and updating of the essential HPT lists (medicines, medical supplies, medical devices, laboratory supplies, equipment, blood and biological products, radiology supplies, nutrition commodities and other health products); •The development and updating of a benefit package for universal health coverage •The negotiation of prices of medicines, vaccines, and medical devices. •The development and updating of a list of essential HPTs for tax exemptions” “Therefore, HTA will inform the decisions of the following organizations: National MOH, The NHIF, Kenya Medical Supplies Agency (KEMSA), The National Treasury” “It is proposed that the following organizational arrangements be established and implemented to support the institutional design for HTA: HTA unit, HTA council in the short term, and HTA agency in the long term, HTA council secretariat, Health Economics Reference Group; Health Services Stakeholder working Groups, Medicines and vaccines stakeholder working groups, Medical devices stakeholder working groups” “Intervention selection will be carried out by explicit priority setting criteria that has been developed by the health benefits advisory panel. These criteria are: Effectiveness and safety, Burden of disease, Severity of disease, Equity, Service, health commodities, and technology requirements, Health workforce requirements, Catastrophic health expenditure, Congruence with existing priorities” “Interventions be subjected to the following assessment: Cost-effectiveness analysis and Budget impact analysis”

*‘We are already seeing HTA in action within the current life of the Kenya Health Policy with the establishment of the panel [HBPAP] and MAPAC’* Participant 1, Development Partner

While policies highlighting institutional and organizational arrangements for HTA exist in Kenya, they do not explicitly indicate sources and amounts of funding to be allocated for HTA-related activities. According to participants and document reviews, all organizational and institutional arrangements for HTA need to be explicitly defined and legislated to support institutionalization of HTA [34].

*‘To institutionalize the proposed HTA process, it is proposed that a HTA policy be developed, and the requirement for HTA in benefit package decision-making be enshrined in the law. This could be in the form of an amendment to the Health Act’* Document excerpt [34]

#### Lack of HTA guidelines and decision-making frameworks

Kenya lacks standardized process and methods guidelines as well as decision-making frameworks for HTA. For example, there were no process guidelines that would inform which rules and procedures would guide the different stages (nomination, selection, assessment, appraisal and decision-making) of the HTA process. There were also no methods guidelines or tools to inform choice of costing perspective and discount rates, or to measure quality adjusted life years. Lastly, there were no decision-making frameworks such as cost-effectiveness threshold to inform decision-making. The lack of HTA guidelines and decision-making frameworks undermined the country’s capacity to conduct and utilize HTA.

*‘We do not have a cost-effectiveness threshold or a quality adjusted life year set for Kenya. We must develop these tools if we are to use HTA routinely for decision-making’* Participant 1, local research organization

### Increasing availability of learning and advocacy for HTA

#### Increasing availability of HTA capacity-building initiatives

Several short-term HTA training workshops and courses have been conducted in Kenya since 2018 [35, 36]. These capacity-building initiatives were targeted at HTA users such as national and county-level policymakers and, HTA doers such as academics and researchers in local universities, research centres, and semi-autonomous government agencies. Approximately 150 HTA doers and users across the health system have been trained on cost-effectiveness analysis and systematic evidence synthesis. According to study participants, these capacity-building initiatives not only built technical capacity but also raised individual and organizational awareness and understanding of the value of HTA in healthcare priority-setting. The initiatives also helped to build a network of champions for HTA.

*‘We have had several workshops which are good for building technical capacity. They also sensitize people to understand the value of HTA. In turn, these people are getting other key stakeholders within the organizations to appreciate what HTA is about.’* Participant 7, Semi-autonomous government agency

A local research organization has also created a mailing list for sending monthly HTA newsletters. These newsletters aim to promote continuous dissemination of HTA knowledge to HTA doers and users.

*‘Every month, we send out a newsletter with interesting topics related to HTA such as economic evaluations, HTA-related conferences, or any forthcoming trainings. We do this to keep HTA relevant on people’s minds.’* Participant 3, local research organization

Despite the increasing availability of short-term capacity-building intiatives, long-term capacity-building initiatives such as undergraduate and postgraduate training in HTA remain limited. This undermined the availability of skilled human resource for HTA. Study participants therefore called for the introduction of HTA-related courses at undergraduate and postgraduate level in public and private universities to strengthen individual and organizational capacity for HTA.

‘*We need more HTA courses in our universities. Their curriculum should be structured to ensure that aspects of health economics and research methodologies such as data analysis and evidence synthesis are captured’* Participant 5, local academic organization

#### Increasing availability of advocacy and awareness creation for HTA

Since 2018, there have been several advocacy and awareness creation initiatives for HTA in Kenya such as study tours, advocacy meetings, and stakeholder engagement workshops. For example, Kenyan stakeholders namely the Parliamentary Health Committee, the Senate Health committee, the MOH, the County Governments, Academics, HBPAP, and the National Health Insurance Fund Health Financing Experts Panel have gone on study tours to Thailand to learn about UHC and the role of HTA in UHC- related decisions [37].

High-level policy advocacy meetings for HTA have also been held in Kenya with participants including officials from the Kingdom of Thailand and Kenyan stakeholders from the Council of Governors, MOH, and National Treasury. There have also been stakeholder engagement workshops for HTA involving policymakers from the MOH and semi-autonomous government agencies. These advocacy meetings and stakeholder engagement workshops were aimed at raising awareness among key policy and decisionmakers on the definition of HTA, its role in healthcare priority-setting processes and policy decisions and, its value in generating budget savings through price negotiations [38–40].

The study tours, high-level policy meetings, and workshops have increased HTA awareness among key policy and decision-makers in government and semi-autonomous government agencies. A landscape assessment of HTA awareness among major health sector agencies at the national level in Kenya conducted in 2020 showed that over 60% of the respondents indicated that the leadership of these agencies were now aware of HTA and were willing to support development of HTA within their organizations by allocating resources [34].

### Stakeholder-related factors

#### Varying stakeholders’ interests towards HTA

##### Policymakers’ interests in UHC and optimal allocation of resources

Participants reported that policymakers’ interest in achieiving UHC and the accompanying need to define a publicly funded health benefits package for the UHC programme drove their support for explicit and evidence-based approaches such as HTA. In addition, policymakers’ interests in allocating scarce health system resources optimally generated further interest in HTA as a tool for informing resource allocation decisions. This need intensified during the Covid-19 pandemic which exposed the inability of Kenya’s health system to meet increased healthcare needs. Consequently, MOH policymakers requested local research organizations to conduct HTA to inform government’s resource allocation decisions during the Covid-19 pandemic such as allocation of oxygen cylinders [41].

*‘COVID not only highlighted but also amplified the gaps in our health system in terms of lack of finances, human resources, infrastructure and medicines. The ministry’s decisions on what to prioritize during the pandemic had to be made systematically using evidence and HTA provided that. It is one of the positive things that Covid did for us’* Participant 4, MOH

##### Technocrats’ interests in evidence-based resource allocation processes

Participants reported that technocrats supported institutionalization of HTA given their interests in evidence-based resource allocation processes. These technocrats included Kenyan health economists and health systems experts within the MOH, local academic and research organizations, and development partners. Technocrats supported HTA as they believed it would provide an evidence-based approach for improving affordability, sustainability, and equitable distribution of health benefits packages in Kenya. They were also responsible for recommending various institutional and organizational arrangements for HTA as presented in the health policies outlined in Table 3.

*‘Health economists and other specialists who sat in committees at the national level were instrumental in designing the content of those policies which catapulted the agenda for transforming the health system through evidence-based processes such as HTA’* Participant 7, MOH

##### Industries’ interests in safeguarding their revenue

In Kenya, lack of price regulation of health technologies has resulted in importer mark-ups ranging between 54 and 256% and 133-748% for generic and originator products respectively [30]. While this generated higher profits for industries and other associated organizations, it led to unaffordability and inequitable access to health technologies. The MOH, through MAPAC, is seeking to use HTA to regulate pricing of health technologies to enable equitable and affordable access. However, according to participants and media reports [42], this was likely to reduce the profit margins for industries and importers of health technologies leading to resistance to institutionalization of HTA.

*‘One barrier will be industries that have been benefitting from the lack of HTA. If we introduce HTA, then they are not going to benefit from the lack of transparency and they are likely to resist’* Participant 1, Semi-autonomous government agency.

#### Limited HTA awareness among officials in county governments and health facilities

Despite growing awareness of HTA among policymakers at the national level, study participants reported that awareness of HTA and its value in policymaking was still low among policy and decision-makers at the county government and hospital levels. These stakeholders were important given Kenya’s devolved health system structure. Their limited awareness was therefore undermining institutionalization of HTA in Kenya. Participants called for greater inclusion of sub-national stakeholders in training and advocacy initiatives to support institutionalization of HTA through greater stakeholder awareness, acceptability, and ownership.

*‘For institutionalization of HTA to take place, we need everyone to buy into HTA starting from the policymakers at the ministry to the frontline workers. For this to work countrywide, counties must be involved. There is need for further sensitization’* Participant 4, Semi-autonomous government agency

### Collaborative support for HTA

#### Presence of international collaboration for HTA

International collaboration for HTA in Kenya occurred through a bilateral agreement between Kenya and Thailand. In February 2019, Kenya and Thailand’s ministries of health signed a bilateral memorandum of understanding on Health Collaboration to support institutionalization of HTA in Kenya [43]. As part of this memorandum, the Kingdom of Thailand- through the Health Intervention and Technology Assessment Program (HITAP) - has provided Kenya’s MOH with technical assistance to develop the HTA institutionalization framework, to build individual and organizational technical capacity for HTA, and to conduct HTA pilot studies of priority to the country. The Kingdom of Thailand has also provided scholarships for HTA at Masters and Doctor of Philosophy level in an effort to promote Kenya’s technical capacity for HTA [43].

*‘The Kenyan government in partnership with the Kingdom of Thailand are working to do a technical transfer between the two countries showing goodwill bilaterally’* Participant 3, MOH

International collaboration for HTA in Kenya also occurred through global health networks such as the International Decision Support Initiative (iDSI). iDSI aims to support low and middle-income countries to reform their healthcare priority setting processes. Since 2019, the iDSI has financially supported several HTA workshops with the aim of building organizational capacity for HTA.

*‘In terms of international efforts, the iDSI has been working with its local partner in Kenya to build capacity for HTA through workshops.’* Participant 6, local research organization

#### Involvement of a bilateral agency

The Japan International Cooperation Agency (JICA)- a bilateral agency- has offered Kenya a conditional grant to support institutionalization of HTA [44]. The disbursements of this conditional grant were tied to specific HTA institutionalization deliverables such as the development of a strategy for HTA institutionalization and capacity building [44]. These loan conditions incentivized Kenya’s MOH to conduct capacity-building workshops and to develop a strategic framework for institutionalizing HTA.

*‘JICA is financing HTA institutionalization efforts in Kenya’* Participant 4, Development partner

## Discussion

In 2018, Kenya introduced a reform on explicit healthcare priority-setting processes that aimed to institutionalize HTA to inform resource allocation decisions within the health sector. Given the limited availability of studies examining institutionalization of HTA in low and lower-middle income countries, we conducted this study to examine the factors that were influencing institutionalization of HTA in Kenya- a lower middle-income country. The key insights derived from this study include the following.

The first key insight was that Kenya’s journey towards institutionalizing HTA was being supported by the following factors: - (a) establishment of organizational structures to conduct, utilize, and/ or oversee HTA; (b) availability of legal frameworks and policies on HTA; (c) increasing availability of awareness creation and capacity-building initiatives for HTA; (d) policymakers’ interests in UHC and optimal allocation of resources; (e) technocrats’ interests in evidence-based processes such as HTA for informing resource allocation; (f) presence of international collaboration for HTA which supported HTA capacity-building and awareness creation activities; and (g) involvement of JICA- a bilateral agency- which supported capacity-building and development of a framework for institutionalization of HTA. The supportive influence of these factors on institutionalization of HTA has been reported in other settings. For example, the establishment of organizational structure(s) expanded the capacity of countries such as Canada [45] and the United Kingdom [46] to conduct and utilize HTA. *Secondly*, the availability of legislation and policies on HTA in Denmark [47], Germany [48] and Thailand [49] supported institutionalization by defining institutional and organization arrangements for HTA. *Thirdly*, the availability of awareness creation activities increased the visibility of the value of HTA to health systems stakeholders in Spain [50] while the availability of short and long-term capacity-building initiatives in Thailand strengthened the human resource capacity for HTA [49, 51]. *Fourthly*, government’s interest in UHC and efficient allocation of resources promoted development of HTA in Netherlands [52, 53]. *Fifthly*, technocrats interests’ in the use of HTA to improve health system performance supported institutionalization of HTA in Mexico [54]. *Sixthly*, international collaboration through iDSI contributed to increased HTA awareness creation and capacity-building initiatives in Indonesia [55], Ghana [56], and South Africa [57]. *Lastly*, involvement of bilateral agencies such as the World Health Organization and the World Bank supported funding of HTA projects and capacity-building initiatives in China [58] and Indonesia [55].

The second key insight was that several factors were undermining Kenya’s journey towards institutionalizing HTA namely: - (a) limited availability of organizational resources such as skilled human resources, financial resources, and information resources for HTA; (b) lack of HTA guidelines and decision-making frameworks; (c) limited HTA awareness among policy and decision-makers at the subnational level- that is, county governments and health facilities; and (d) industries’ interests in safeguarding their revenue. The limiting influence of these factors on institutionalization of HTA has been reported in other settings. For example, limited availability of skilled human resource for HTA undermined capacity to conduct HTA in India [59], Iran [60], South Africa [61] and Tanzania [62]. *Secondly*, limited availability of financial resources undermined institutionalization of HTA in Iran [63], South Africa [61] and Tanzania [62]. *Thirdly*, limited availability and completeness of data for HTA undermined institutionalization of HTA in several high, middle and low-income countries globally [15, 17]. *Fourthly*, the lack or limited availability of contextually relevant process and methodological guidelines, and decision tools has undermined utilization of HTA in Sub Saharan countries [11]. *Fifthly*, the limited awareness of HTA, its concepts and relevance among policy and decision-makers in Malaysia [64] and South Africa [61] undermined institutionalization of HTA. *Lastly*, manufacturers’ interests in safeguarding pricing of their health technologies undermined institutionalization of HTA in the United States of America [65, 66].

The third key insight was that some of the factors that were influencing institutionalization of HTA in Kenya were interlinked. These interlinkages have also been reported in other studies. For example, international collaboration for HTA increased the availability of HTA awareness creation and capacity-building activities in Kenya. Literature shows that countries involved in international collaborative networks across Europe and Asia reported similar findings [67, 68]. Secondly, the interests of Kenyan policymakers in defining a health benefits package for UHC and in regulating pricing of health technologies led to the creation of several organizational structures for HTA in Kenya such as HBPAP and MAPAC. Similar findings have also been reported in several high and upper-middle-income countries in Asia [51, 69] and Europe [52, 53] where policymakers’ interests in UHC and regulation of pricing led to the establishment of HTA agencies. Thirdly, availability of legislation and policies on HTA partly led to the creation of organizational structures for HTA in Kenya. Several high-income countries in Europe with legislation and policies on HTA reported similar findings [68]. Fourthly, technocrats’ interests in HTA influenced development of policies on HTA in Kenya. Similarly, technocrats in several high- and middle-income countries in Asia developed policies on HTA due to their interests in HTA [64, 70]. Lastly, the limited availability of long-term capacity-building initiatives undermined the availability of skilled human resource for HTA in Kenya. Similar findings have been reported in other countries globally with limited availability of long-term capacity-building initiatives [17, 18, 61, 71].

The study findings offer important policy implications on how the MOH can nurture and sustain the institutionalization process in Kenya. This can be achieved through a systemic approach that addresses the current limitations in Kenya’s capacity to conduct and utilize HTA. In this systemic approach, the MOH should earmark funds from the national health budget to ensure adequate availability of financial resources for HTA. The MOH should introduce a cost database and promote timely data collection through trainings and incentives to strengthen information resources for HTA. The MOH should collaborate with academic institutions offering health sector focused undergraduate and graduate training (e.g., medical schools and schools of public health) to introduce and integrate HTA training in their undergraduate and postgraduate courses to ensure availability of skilled human resource for HTA. The MOH should develop contextually relevant process and methods guidelines and decision tools for HTA to facilitate how HTA processes are conducted and how HTA outputs are utilized in decision-making. The MOH should conduct wider advocacy to increase HTA awareness among national and sub-national stakeholders. The MOH should manage stakeholders’ interests through sensitization and persuasive framing of the value of institutionalizing HTA to minimize opposition. Lastly, the MOH should strengthen international collaborations through south-south collaborations to facilitate the institutionalization process.

Lastly, by applying the conceptual framework outlined in this study (Fig. 1), we demonstrated its empirical utility, that is, the framework was comprehensible and applicable practically in identifying factors that were influencing institutionalization of HTA in Kenya. In addition, we found that there were interlinkages between the factors just as indicated in the conceptual framework. This framework therefore provides a simple and comprehensive approach for systematically identifying factors that influence institutionalization of HTA in a health system. Future researchers can adopt or adapt this framework to examine factors influencing institutionalization of HTA in other contexts while recognizing the possibility of interlinkages between the factors.

### Limitations

A potential limitation of this study is social desirability bias whereby participants alter responses in the belief that this would make the responses more acceptable. However, by triangulating data sources and methods, we strengthened the trustworthiness of the findings. It is also possible that the previous involvement of 2 of the authors in HTA-related processes in Kenya may have biased the interviews and analysis. However, we mitigated against this bias by reviewing documents to corroborate the findings and by holding peer debriefing sessions as a study team to ensure that findings were based on collected data. Lastly, a potential limitation of this study is the absence of patient advocacy or patient representatives in the sample population. Future studies should consider incorporating the voice of this sampling population.

## Conclusion

Examining factors that influence institutionalization of HTA is substantially relevant in low and middle-income countries where institutionalization of HTA remains limited. In this study, we used a conceptual framework based on five sets of factors that were identified from a scoping review on factors influencing institutionalization of HTA across countries of different income levels. By applying this conceptual framework, we were able to identify factors that were supporting and limiting institutionalization of HTA in Kenya. These findings offer useful policy implications that policymakers within the MOH can implement to facilitate progress towards institutionalization of HTA in Kenya. Researchers and policymakers can use this study’s conceptual framework to examine factors influencing institutionalization of HTA or to develop a roadmap for institutionalizing HTA in contexts of interest respectively.

## Electronic supplementary material

Below is the link to the electronic supplementary material.

Supplementary Material 1

## Data Availability

The datasets generated and/or analysed during the current study are not publicly available due ethical reasons that include maintaining participants’ confidentiality and anonymity but are available from the corresponding author on reasonable request.

## List of abbreviations

HBPAP: Health Benefits Package Advisory Panel
HTA: Health Technology Assessment
iDSI: International Decision Support Initiative
JICA: Japan International Cooperation Agency
MAPAC: Medicines Affordability Pricing Advisory Committee
MOH: Ministry of Health
UHC: Universal Health Coverage

## References

[1] Chalkidou K, Glassman A, Marten R, Vega J, Teerawattananon Y, Tritasavit N, Gyansa-Lutterodt M, Seiter A, Kieny MP, Hofman K (2016). Priority-setting for achieving universal health coverage. Bull World Health Organ.

[2] World Health Organization: Making fair choices on the path to universal health coverage. Final report of the WHO consultative group on equity and universal health coverage. In. Geneva, Switzerland. ; 2014: 1–84.

[3] O’Rourke B, Oortwijn W, Schuller T (2020). The new definition of health technology assessment: a milestone in international collaboration. Int J Technol Assess Health Care.

[4] World Health Assembly. Sixtieth World Health Assembly- WHA 60.29. Health Technologies. World Health Organization; 2007. pp. 1–2. https://apps.who.int/iris/handle/10665/22609

[5] Norheim OF (2015). The elusive challenge of priority setting in health and health care. Global Challenges.

[6] Evans TG, Palu T (2016). Setting Priorities, Building Prosperity through Universal Health Coverage. Health Syst Reform.

[7] World Health Assembly. Health intervention and technology assessment in support of universal health coverage (Resolution WHA 67.23). World Health Organization; 2014. http://apps.who.int/gb/ebwha/pdf_files/wha67/a67_r23-en.pdf

[8] World Health Organization. : Health technology assessment of medical devices. WHO Medical devices technical series. In. https://apps.who.int/iris/handle/10665/44564: World Health Organization 2011: 1–44.

[9] Bertram M, Dhaene G, Tan-Torres Edejer T. Institutionalizing Health Technology Assessment Mechanisms: a how to Guide. World Health Organization; 2021. pp. 1–66. https://apps.who.int/iris/handle/10665/340722

[10] World Health Organization. Institutionalization of Health Technology Assessment. World Health Organization Regional Office for Europe; 2001. pp. 1–27. https://apps.who.int/iris/bitstream/handle/10665/108382/E72364.pdf?sequence=1&isAllowed=y

[11] Hollingworth S, Fenny AP, Yu S-Y, Ruiz F, Chalkidou K (2021). Health technology assessment in sub-saharan Africa: a descriptive analysis and narrative synthesis. Cost Eff Resource Allocation.

[12] Chalkidou K, Li R, Culyer AJ, Glassman A, Hofman KJ, Teerawattananon Y (2017). Health technology assessment: global advocacy and local realities: comment on” priority setting for universal health coverage: we need evidence-informed deliberative processes, not just more evidence on cost-effectiveness. Int J Health Policy Manage.

[13] Suharlim C, Kumar R, Salim J, Mehra M, Gilmartin C, Caruso AA, Castro H (2022). Exploring facilitators and barriers to introducing health technology assessment: a systematic review. Int J Technol Assess Health Care.

[14] Kaló Z, Gheorghe A, Huic M, Csanádi M, Kristensen FB (2016). HTA implementation roadmap in Central and Eastern European countries. Health Econ.

[15] Rajan A, Gutierrez-Ibarluzea I, Moharra M (2011). Addressing issues in health technology assessment promotion: motives, enablers, and barriers. Int J Technol Assess Health Care.

[16] Mbau R, Vassall A, Gilson L, Barasa E. Factors influencing institutionalization of health technology assessment: a scoping literature review. Health Syst Reform Undergoing review:30. 10.1080/23288604.2024.2360315 39158224

[17] World Health Organization. : Global survey on health technology assessment by national authorities. Main Find 2015:1–40.

[18] Babigumira JB, Jenny AM, Bartlein R, Stergachis A, Garrison LP (2016). Health technology assessment in low-and middle-income countries: a landscape assessment. J Pharm Health Serv Res.

[19] The World Bank. Population total- Kenya. The World Bank Group; 2022. https://data.worldbank.org/indicator/SP.POP.TOTL?locations=KE

[20] The Republic of Kenya. The Constitution of Kenya. National Council for Law Reporting; 2010. pp. 1–194. http://kenyalaw.org/lex/actview.xql?actid=Const2010

[21] Saunders B, Sim J, Kingstone T, Baker S, Waterfield J, Bartlam B, Burroughs H, Jinks C (2018). Saturation in qualitative research: exploring its conceptualization and operationalization. Qual Quant.

[22] Braun V, Clarke V (2006). Using thematic analysis in psychology. Qualitative Res Psychol.

[23] Shenton AK (2004). Strategies for ensuring trustworthiness in qualitative research projects. Educ Inform.

[24] Long T, Johnson M (2000). Rigour, reliability and validity in qualitative research. Clin Eff Nurs.

[25] Althubaiti A (2016). Information bias in health research: definition, pitfalls, and adjustment methods. J multidisciplinary Healthc.

[26] Bowen GA (2009). Document analysis as a qualitative research method. Qualitative Res J.

[27] The Executive Office of the President. : The Big 4 agenda: Fasttracking our vision through a 5-year development plan under 4 key pillars. In. https://big4.delivery.go.ke/: Government of Kenya; 2017.

[28] The National Council of Law Reporting (Kenya Law). Authority of the Republic of Kenya (2018). Advisory Panel for the Design and Assessment of the Kenya UHC essential Benefit Package (UHC-EBP). The Kenya Gazette: Gazette Notice No 5627.

[29] Health Benefits Package Advisory Panel: Final Report of the Universal Health Coverage Health Benefits Package Advisory Panel. June 2020. In. Edited by Health Mo. Nairobi: Ministry of Health. ; 2020: 1–25.

[30] Medicines Affordability Pricing Advisory Committee: HTA framework sensitization meeting. In. Nairobi: MAPAC. ; 2021: 1–6.

[31] Ministry of Health: Appointment to the Technical Working Group (TWG) on the Finalization of the Kenya Health Technology Assessment (HTA) Strategy. In. Edited by Office of the Principal Secretary MoH. Nairobi, Kenya.: Ministry of Health. ; 2021: 1–2.

[32] Barasa E, Orangi S, Mbau R, Kairu A. Situational Analysis and Capacity Assessment of Health Technology Assessment (HTA) in Kenya. In. Nairobi: KEMRI-Wellcome Trust; 2021: 1–15.

[33] Ministry of Health. : Health Products and Technologies Supply Chain Strategy 2020–2025. In. https://www.health.go.ke/wp-content/uploads/2020/12/HPT-Supply-ChainStrategy-2020-2025.pdf: Republic of Kenya; 2020: 1-156.

[34] Ministry of Health. Strategy for Health Technology Assessment in the kenyan Health Sector. September 2021. In. Nairobi, Kenya. Ministry of Health; 2021. pp. 1–30.

[35] KEMRI-Wellcome Trust (2019). Report of the Health Technology Short Course held at the Royal Tulip in Nairobi, Kenya from 18th to 22nd November 2019.

[36] Ministry of Health. Government launches health technology assessment to inform policy decision making. Republic of Kenya; 2018. https://www.health.go.ke/government-launches-health-technology-assessment-to-inform-policy-decision-making-nairobi-kenya-18-march-2018/

[37] HITAP. Strenghten collaboration on health between Thailand and Kenya. Health Intervention and Technology Assessment Program, Ministry of Public Health; 2019. https://www.hitap.net/en/news/176667

[38] HITAP: National Hospital Insurance Fund Health Financing Reforms Experts Panel visit to Thailand on Universal Health Coverage (UHC) and. Health Technology Assessment (HTA). HITAP; 2019. pp. 1–32. http://www.globalhitap.net/wp-content/uploads/2020/02/Mission-Report-NHIF-Panel-Visit-to-Thailand-June-2019-ForPublication.pdf

[39] HITAP: Kenya’s Health Benefits Advisory Panel [HBAP] (2018). Study visit to Thailand.

[40] KEMRI-Wellcome Trust: Study visit by the Health Benefits Package Advisory Panel to Thailand on Health Technology Assessment. In. Nairobi: KEMRI-Wellcome Trust Research Programme. ; 2018: 1–23.

[41] Ministry of Health. Press statement on Covid-19. Ministry of Health; 2021. p. 2. https://www.health.go.ke/wp-content/uploads/2021/08/Press-Statement-on-Covid-19-2nd-July-2021-1.pdf

[42] Fitch Solutions: Reference pricing likely to be detrimental to drugmakers’ revenues in Kenya. In., vol. 2022. https://www.fitchsolutions.com/pharma-healthcare/reference-pricing-likely-be-detrimental-drugmakers-revenues-kenya-08-11-2019: Fitch Solutions Country Risk and Industry Research; 2019.

[43] HITAP. Deepening a newly formalized collaboration between Kenya’s Ministry of Health and Thailand’s Ministry of Public Health. Health Intervention and Technology Assessment Program, Ministry of Public Health; 2019. https://www.hitap.net/en/176469

[44] Ministry of Health (2019). JICA Yen Loan Policy Action on HTA.

[45] Battista RN, Côté B, Hodge MJ, Husereau D (2009). Health technology assessment in Canada. Int J Technol Assess Health Care.

[46] Raftery J, Powell J (2013). Health technology assessment in the UK. The Lancet.

[47] Sigmund H, Kristensen FB (2009). Health technology assessment in Denmark: strategy, implementation, and developments. Int J Technol Assess Health Care.

[48] Perleth M, Gibis B, Gohlen B (2009). A short history of health technology assessment in Germany. Int J Technol Assess Health Care.

[49] Teerawattananon Y, Tantivess S, Yothasamut J, Kingkaew P, Chaisiri K (2009). Historical development of health technology assessment in Thailand. Int J Technol Assess Health Care.

[50] Sampietro-Colom L, Asua J, Briones E, Gol J (2009). History of health technology assessment: Spain. Int J Technol Assess Health Care.

[51] Leelahavarong P, Doungthipsirikul S, Kumluang S, Poonchai A, Kittiratchakool N, Chinnacom D, Suchonwanich N, Tantivess S (2019). Health Technology Assessment in Thailand: institutionalization and contribution to Healthcare decision making: review of literature. Int J Technol Assess Health Care.

[52] Chinitz D (2004). Health technology assessment in four countries: response from political science. Int J Technol Assess Health Care.

[53] Bos M (2000). Health technology assessment in the Netherlands. Int J Technol Assess Health Care.

[54] Gómez-Dantés O, Frenk J (2009). Health technology assessment in Mexico. Int J Technol Assess Health Care.

[55] Sharma M, Teerawattananon Y, Luz A, Li R, Rattanavipapong W, Dabak S (2020). Institutionalizing evidence-informed priority setting for universal health coverage: lessons from Indonesia. INQUIRY: The Journal of Health Care Organization Provision and Financing.

[56] Addo R, Hall J, Haas M, Goodall S (2020). The knowledge and attitude of ghanaian decision-makers and researchers towards health technology assessment. Soc Sci Med.

[57] MacQuilkan K, Baker P, Downey L, Ruiz F, Chalkidou K, Prinja S, Zhao K, Wilkinson T, Glassman A, Hofman K (2018). Strengthening health technology assessment systems in the global south: a comparative analysis of the HTA journeys of China, India and South Africa. Global Health Action.

[58] Chen Y, Banta D, Tang Z (2009). Health technology assessment development in China. Int J Technol Assess Health Care.

[59] Jain B, Hiligsmann M, Mathew JL, Evers SM (2014). Analysis of a small group of stakeholders regarding advancing health technology assessment in India. Value in Health Regional Issues.

[60] Arab-Zozani M, Sokhanvar M, Kakemam E, Didehban T, Hassanipour S (2020). History of health technology assessment in Iran. Int J Technol Assess Health Care.

[61] Mueller D (2020). Addressing the challenges of implementing a Health Technology Assessment Policy framework in South Africa. Int J Technol Assess Health Care.

[62] Surgey G, Chalkidou K, Reuben W, Suleman F, Miot J, Hofman K (2019). Introducing health technology assessment in Tanzania. Int J Technol Assess Health Care.

[63] Mohtasham F, Yazdizadeh B, Zali Z, Majdzadeh R, Nedjat S (2016). Health technology assessment in Iran: barriers and solutions. Med J Islamic Repub Iran.

[64] Sivalal S (2009). Health technology assessment in the Asia Pacific region. Int J Technol Assess Health Care.

[65] Callahan D (2012). Health technology assessment implementation: the politics of ethics. Soc Med Decis Mak.

[66] Luce B, Cohen RS (2009). Health technology assessment in the United States. Int J Technol Assess Health Care.

[67] Liu G, Wu EQ, Ahn J, Kamae I, Xie J, Yang H (2020). The development of health technology assessment in Asia: current status and future trends. Value in Health Regional Issues.

[68] Banta D, Kristensen FB, Jonsson E (2009). A history of health technology assessment at the european level. Int J Technol Assess Health Care.

[69] Pwee KH (2009). Health technology assessment in Singapore. Int J Technol Assess Health Care.

[70] Hisashige A (2009). History of healthcare technology assessment in Japan. Int J Technol Assess Health Care.

[71] Jaramillo HEC, Moreno-Mattar O, Osorio-Cuevas D (2016). Emergence of “drivers” for the implementation of health technology assessment. Int J Technol Assess Health Care.

