# Supplementary material for: Factors influencing institutionalization of health technology assessment in Kenya
Source: BMC Health Serv Res. 2023 Jun 22;23:681. doi: 10.1186/s12913-023-09673-4 (PMC10288787; doi:10.1186/s12913-023-09673-4)
Supplement: Supplementary file 1 — Supplementary Material 1 [file 12913_2023_9673_MOESM1_ESM.docx]

**Additional File 1: Interview topic guide**

1. What factors are supporting or hindering institutionalization of HTA in Kenya?

Probes: -

1. *How would you describe the availability and influence of organizational resources (e.g., human resources, financial resources, information resources) on institutionalization of HTA in Kenya?*
2. *How would you describe the availability and influence of legal frameworks and policies on institutionalization of HTA in Kenya?*
3. *How would you describe the availability and influence of capacity building initiatives on institutionalization of HTA in Kenya?*
4. *How would you describe the availability and influence of HTA awareness creation activities in Kenya?*
5. *How would you describe the availability and influence of collaborative support (e.g., international collaboration, involvement of bilateral and multi-lateral agencies) on institutionalization of HTA in Kenya?*
6. *How would you describe the influence of stakeholders’ interests and awareness on institutionalization of HTA in Kenya?*
7. *Are there any other factors that are influencing institutionalization of HTA in Kenya*

2. How can the Ministry of Health support institutionalization of HTA in Kenya?
